# Individual Subject Meta-Analysis of Parameters for *Giardia duodenalis* Shedding in Animal Experimental Models

**DOI:** 10.1155/2014/476142

**Published:** 2014-04-01

**Authors:** A. D. Adell, W. A. Miller, D. J. Harvey, E. Van Wormer, S. Wuertz, P. A. Conrad

**Affiliations:** ^1^Department of Pathology, Microbiology and Immunology, School of Veterinary Medicine, One Shields Avenue, University of California, Davis, CA 95616, USA; ^2^Escuela de Medicina Veterinaria, Facultad de Ecologia y Recursos Naturales, Universidad Andres Bello, Republica 440, 8370251 Santiago, Chile; ^3^One Health Institute, School of Veterinary Medicine, University of California, Davis, CA 95616, USA; ^4^Department of Public Health Sciences, School of Medicine, University of California, Davis, CA 95616, USA; ^5^Department of Civil & Environmental Engineering, College of Engineering, University of California, Davis, CA 95616, USA; ^6^Singapore Centre on Environmental Life Sciences Engineering (SCELSE), School of Biological Sciences, Nanyang Technological University, 60 Nanyang Drive, Singapore 637551; ^7^School of Civil and Environmental Engineering, Nanyang Technological University, 60 Nanyang Drive, Singapore 637551

## Abstract

*Giardia duodenalis* is a zoonotic protozoan parasite with public health importance worldwide. While articles about animal model infectivity have been published for *G. duodenalis*, the studies have used diverse protocols and parameters to evaluate the infectivity of this protozoan parasite. Hence, the objectives of this study were to (1) conduct a meta-analysis of published literature for cyst shedding and diarrhea outcomes in animal models and (2) develop recommendations to help standardize experimental dose response studies. Results showed that, for the outcome of cyst shedding in faeces, the covariates of infective stage (cyst versus trophozoite), *Giardia* dose, and the interactions between doses and infective stage, as well as dose and species of experimental host, were all significant (*P* value ≤ 0.05). This study suggests inoculation of the experimental host with cysts rather than trophozoites and administration of higher doses of *Giardia* will most likely result in cyst shedding. Based on the results of this meta-analysis, the infective stage (cyst versus trophozoite), parasite dose, and the interactions between dose and infective stage, as well as dose and species of experimental host, should be considered when designing experimental dose response studies that will assist in the study of zoonotic neglected tropical diseases globally.

## 1. Introduction

*Giardia duodenalis* is a zoonotic waterborne pathogen known to be released into the environment through human and animal faeces [1]. Infection and gastrointestinal disease in humans are caused by ingestion of small doses (>10 cysts) of* Giardia* cysts [2] directly from faeces or indirectly from contaminated water and food products [1, 3].* Giardia *is considered the most common cause of protozoan diarrhea in developing countries and worldwide [1]. It has been estimated that in Africa, Asia, and Latin America about 200 million people have symptomatic giardiasis with some 500000 new cases being reported each year [4]. The number of reported cases in the US was 18,913 in 2008 [5]. In 2004,* Giardia *was included in the World Health Organization's Neglected Disease Initiative for its link with poverty [6].* G. duodenalis, *also called* G. intestinalis* and* G. lamblia, *is the only species found in humans [1, 3] and will be referred to as* G. duodenalis *throughout this paper.

Animal and human dose-response models as well as in vitro cell culture have been widely used to evaluate* Giardia *spp. infectivity. At present, human infectivity studies are not considered to be practical because of the ethical concerns involved. Therefore, very few experiments have involved the attempted infection of humans with* Giardia* isolates [2, 7]. Instead, experimental animal models have been frequently used to estimate whether* G. duodenalis* is strictly host specific or zoonotic [1] and to provide information used for human health risk assessments. In vitro cell culture has been proposed as a potential alternative to animal assays because cultivation has the advantage of being cheaper and less time consuming and does not raise the ethical concerns associated with using animal models [8]. However, experimental animal model studies are often preferred when performing risk assessment, as they provide the most realistic scenario of what is happening with the pathogen once it enters the host. For this reason, this meta-analysis focuses on the diverse range of experimental animal model studies available in the peer-reviewed literature.

Numerous cross-transmission experiments involving the infection with isolates of* Giardia* in a variety of animal species have been published. Each of the experimental studies used different parameters such as animal species, age, cysts or trophozoites, dose, and detection methods to quantify cysts in faeces and evaluate the infectivity of* G. duodenalis* as well as outcomes ranging from laboratory detection of cyst shedding to clinical symptoms. Using diverse variables and protocols can be problematic in experimental studies because the variations in protocols between studies can affect the outcome of the experiments and thus their comparability [1, 9]. Consequently, a standardization of the experimental dose-response studies is required.

Hence the main objective of the present study was to develop recommendations for the standardization of animal dose-response experiments by conducting a meta-analysis of individual subjects and exploring study design characteristics that cause heterogeneity between included studies. The hypothesis tested in this meta-analysis was that selected experimental factors of interest are associated with increased* G. duodenalis* cyst shedding and diarrhoea. The results of this meta-analysis will be useful for more standardized and comparable studies of* Giardia* infectivity, so that in the future there may be fewer animal species and resources used to provide dose-response information more quickly and efficiently.

## 2. Methods

### 2.1. Literature Search Strategy and Selection of Studies

**Figure 1:**
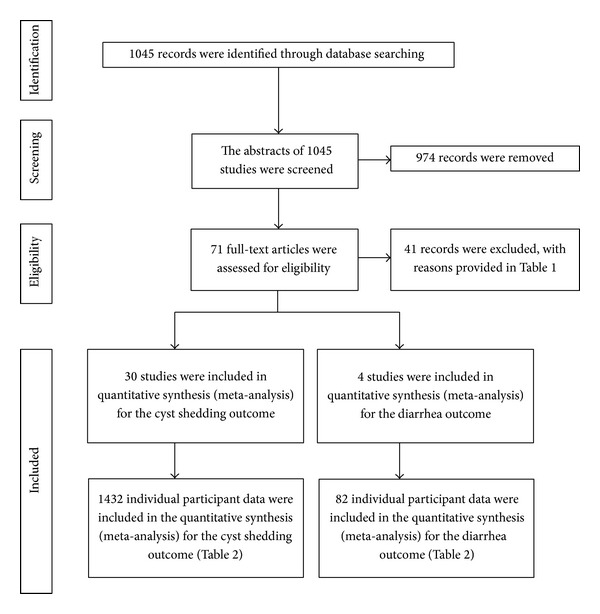
Flow of information through the different phases of a systematic literature review for the* Giardia duodenalis* meta-analysis.

The search of published animal and/or human dose-response studies in all languages was performed using the electronic databases PubMed and Web of Science from March to December, 2010. The process is described in Figure 1, with search criteria consisting of the following algorithms: (i) protozoan infectious doses for humans, (ii)* Giardia* and dose response, (iii)* Giardia* and infectivity, (iv)* Giardia* and meta-analysis, (v) giardiasis and experimental and model, and (vi) experimental and infection and* Giardia*. Unpublished studies (grey literature) were not included in this meta-analysis.

The articles identified by means of these search criteria were subject to a further selection process consisting of the removal of the complete study or individuals within the articles that met any of the exclusion criteria (Table 1). One reviewer examined the titles and abstracts of all the articles found using the search criteria mentioned above. The full article for each of the relevant studies was then assessed by the same reviewer and a second reviewer was consulted as needed. Only those articles that met at least one of the search criteria and none of the exclusion criteria were included in the data analysis. Furthermore, the reference lists of all included articles were searched for further possible papers, but no additions were identified. Ethical approval was not required for this meta-analysis.

### 2.2. Data Extraction

Data regarding the following variables for each individual animal were extracted and recorded from each article: (i)* Giardia species* (*Giardia*), (ii) isolate analyzed, such as HP-10 (Isolate), (iii) assemblage of the* Giardia sp*. such as A (Assemblage), (iv) original source of isolate (Isolate Source), (v) whether cysts or trophozoites were subject to passage before challenge (Passage), (vi) storage time of cysts or trophozoites (in weeks) prior to inoculation of experimental host (Storage Time), (vii) method used to confirm cyst or trophozoite viability prior to inoculation (Viability Method), (viii) animal species used as experimental host (Experimental Species), (ix) number of subjects per group inoculated (Number), (x) age of the experimental host (Age), (xi) whether the experimental host was subject to immunosuppression (Immunosuppression), (xii) method of immunosuppression of the experimental host (Immunosuppression Cause), (xiii) whether cysts or trophozoites were administered to the experimental host (Infective Stage), (xiv) cyst or trophozoite dose administered to the experimental host (*Giardia* Dose), (xv) administration route used to inoculate the cysts or trophozoite dose to the experimental host (Administration Route), (xvi) cyst detection method in faeces of experimental hosts after inoculation (Detection Method), and (xvii) the number of animals that shed cysts and/or presented with diarrhoea after inoculation.

The unit of analysis was the individual animal; thus, the value for each variable was collected for each animal and included in the analysis. If a variable was not reported at the animal level, the variable was left blank.  Table 2 provides information regarding the number of subjects and missing values by variable and study. The primary outcomes of interest were the presence of cyst shedding and diarrhoea. An animal was considered to have shedding or diarrhoea when the condition of the animal was described with the words diarrhoea or cyst shedding in the study from which the information was extracted. All of the studies included in this analysis evaluated shedding over prolonged periods so even if shedding was intermittent, any report of cysts observed in the feces was considered as positive for shedding. For diarrhoea and cyst shedding, the classification of Yes/No was used. Initially, attempts were made to contact authors for clarification, but, due to lack of responses, this approach was not systematically implemented throughout the entire study.

### 2.3. Classification of Variables

The extracted variables were classified according to the information provided by the selected studies for each outcome (Table 3). In the case of the variable “Isolate Source,” the classification “Other than Humans” was composed of ruminants (cattle and lambs), primates, rodents, and drinking water. For the variable “Passages,” the classification “Yes” was composed of in vivo (animal passage), in vitro (cultures), and both in vivo and in vitro passages, while the classification “No” corresponded to cysts that were not subject to any passage. For the “Storage Time” variable, the class “<1 week” included animals that were inoculated with cysts or trophozoites that were stored for less than one week; “≥1 week” included all those animals that were inoculated with cysts or trophozoites that were stored for one week or more. Treating the variable “Storage Time” as a continuous variable, with or without log transformation, did not improve the model fit. Regarding the variable “Experimental Species,” the category “Other Animals” included dogs, cats, and cattle, while the class “Other Rodents” comprised rats and hamsters. The variable “Age” was categorized following published criteria (Table 4), which were consistent with the criteria used in the studies included in this meta-analysis. The classification of age was dependent on the animal model as animal species mature at different rates. For instance, 2-month-old kittens are in a different maturity stage than 2-month-old gerbils and thus might have different susceptibilities to protozoan infection. When ranges of age were reported, the mean of the range was used in the meta-analysis. The classification “Young” in the “Age” variable for the shedding outcome consisted of newborn and weanling animals, while the classification “Adult” included only adult animals. For the diarrhoea outcome, no adult animals with diarrhoea were reported. Therefore, the “Age” variable for the diarrhoea outcome was composed of “Newborn” and “Weanling.” For the variable “Administration Route,” “Gastric Intubation” included the terms gastric intubation, intragastric route, and stomach tube, while “Other Than Gastric Intubation” included the terms orally, intraesophageal route, and nasogastric. The variable “Detection Method” was only included in the cyst shedding outcome and included three classes: “Hemocytometer,” “Microscopy,” and “Flotation Techniques.” “Microscopy” included indirect fluorescent antibody (IFA), fluorescent microscopy, and Nomarski interference contrast (NIC) microscopy. “Flotation Technique” included parasite concentration methods such as zinc sulfate flotation and sucrose gradient flotation. The variable “*Giardia* Dose” was a continuous variable. As the values in the “*Giardia* Dose” variable ranged from 4 to 10^7^ cysts or trophozoites, the data for this variable were log transformed to make the distribution of the data more normal. The mean of the whole dose series (mean log scale = 4.12) was subtracted from every dose value to center the data. This enabled interpretation of the main effect in the presence of an interaction term at a meaningful value (the mean) [10]. For instance, in the multivariable model, the main effect of “Infective Stage” in the model that also includes the interaction “*Giardia *Dose by Infective Stage” corresponded to a difference between cysts and trophozoites at a mean dose. If the mean of the whole dose series was not subtracted, this comparison would have been the difference between cysts and trophozoites at a log dose of zero (dose of 1).

### 2.4. Statistical Analyses

In this meta-analysis, the statistical analysis and publication bias assessment were done as described in Adell et al. [11]. Final models were selected based on inclusion of the four key variables “Experimental Species,” “Age,” “*Giardia* Dose,” and “Infective Stage,” allowing for as many interaction terms as possible and having a combination of variables that generated narrower confidence intervals or more precision. The fit of the model was assessed by the ratio of the generalized chi-square statistic and its degrees of freedom (generalized chi-sq/df), which could not exceed a value of 1, and residual plots. The statistical software JMP 9 (SAS 2010) and SAS 9.3 (SAS 2011) were used to perform all the analyses.

## 3. Results

### 3.1. Search Strategy and Selection of Studies

As shown in Figure 1, the initial search identified 1045 potentially relevant studies. Subsequent to reviewing the abstracts, 71 studies were considered for further screening, of which 41 were not eligible since at least one of the exclusion criteria (Table 1) was met. After examination of the full text, 30 studies were included for the cyst shedding outcome while 4 were incorporated for the diarrhoea outcome. The studies included in this meta-analysis were published between 1978 and 2010 and are shown in Table 2. The total number of individuals included in this meta-analysis was 1432 individuals for the shedding outcome and 82 individuals for the diarrhoea outcome.

### 3.2. Analysis for the Cyst Shedding Outcome

The bivariate analysis showed that the covariates “Passage,” “Experimental Species,” “Age,” “Infective Stage,” and “*Giardia* Dose” were associated with cyst shedding in faeces (*P* value ≤ 0.2) (Table 5). These variables were then incorporated in the multivariable analysis which provided one model that best fulfilled the selection criteria. The final multivariable model (Table 6) shows that the variables “Infective Stage” and “*Giardia* Dose” and the interactions “*Giardia* Dose by Experimental Species” and “*Giardia* Dose by Infective Stage” have at least one category with a statistically significant difference (*P* value ≤ 0.05) from the reference category (Table 6). The variables “Experimental Species,” “Age,” and “Administration Route” were not significant, but they were incorporated into the model to control for confounding and effect modification. “Administration route” and “Age” were identified as a potential confounder (an epidemiologic term specifically describing a variable associated with a change in the coefficient estimate of at least one variable when placed in the model) of the relationship between “*Giardia *dose” and cyst shedding and “Experimental Species” and cysts shedding, respectively. “Experimental species” was part of the interactions “*Giardia* Dose by Experimental Species.”

The multivariable model indicated that inoculating cysts into the experimental host had 5.02 times higher odds of cyst shedding than inoculating trophozoites at the mean log dose of 0.0538 (*P* value ≤ 0.001; CI: 2.63, 9.56). For each 1 unit of change in the log of trophozoite dose administered to the reference experimental host “Gerbil,” the odds of cyst shedding increased 2.67 times (*P* value < 0.0001; CI: 1.81, 3.94), whilst the odds ratio corresponding to an increase of one in log dose in cysts was 0.57 times that in trophozoites (*P* value = 0.002; CI: 0.41, 0.81).

**Figure 2:**
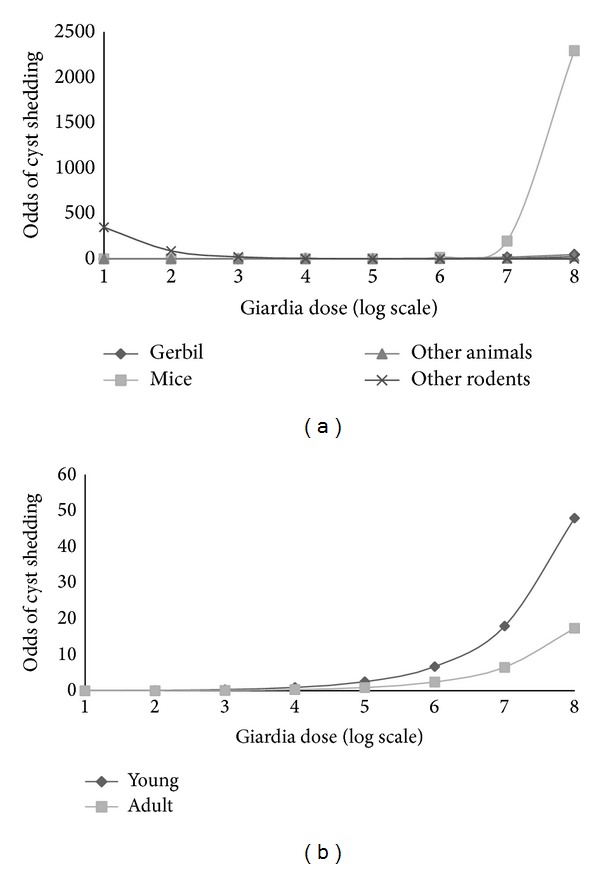
Range of* Giardia* dosages captured in the articles included in this meta-analysis by the odds of the cyst shedding outcome for the categories “Age” and “Experimental Species.” (a) Range of* Giardia* dosages (log scale) by odds of cyst shedding by Experimental Species. (b) Range of* Giardia* dosages (log scale) by odds of cyst shedding by Age.

The model also indicated that, for each unit of increase in the log dose administered to “Mice,” the odds of cyst shedding increased 4.36 times compared to “Gerbils” (*P* value: 0.004; CI: 1.59, 11.93). Whereas, for each unit increase in the log dose administered to “Other Rodents,” the odds of having cyst shedding were reduced 0.09 times compared to “Gerbils” (*P* value: 0.02; CI: 0.01, 0.72).  Figure 2(a) shows the range of doses compared to the odds of detecting shedding by experimental species, illustrating how the choice of the experimental animal species does not have an effect until beyond a log scale 6, with mice only having an increase in odds. When “Mice” was removed from the analysis, the odds of shedding cysts were similar for all animals until log 7 after which the odds of shedding were greater in “Gerbils” as opposed to “Other animals.” Figure 2(b) shows the ranges of* Giardia *doses compared to the odds of detecting shedding and how choosing young experimental animals had a higher impact on shedding in response to different doses being investigated compared to adult experimental animal.

The fit of the model was assessed by the ratio value for generalized chi-sq/df value and residual plots. The generalized chi-sq/df for the multivariable model was 0.66, indicating that the model had good fit. The residual plots (not shown), in which the residual values were plotted against the linear predictor, showed that two observations in each model had large residuals (≥15) and were possible outliers from the cluster of observations. Removing the individuals that had large residuals did not change the significance of the other variables of the model; thus, they were kept in the model.

**Figure 3:**
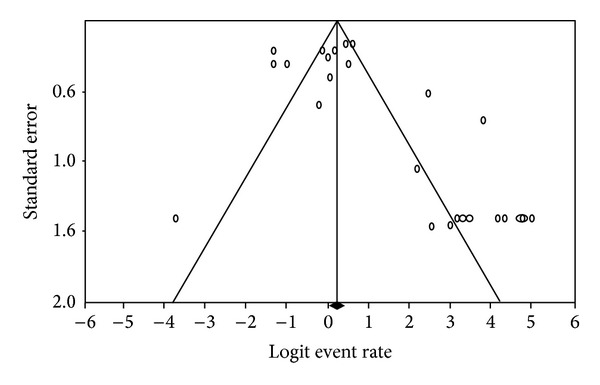
Funnel plot of standard error by logit event rate for cyst shedding outcome after* Giardia* infection showing possible publication bias as evidenced by a higher concentration of studies to the right of the mean.

In this meta-analysis, we found evidence of possible publication bias for the cyst shedding outcome. The funnel plot showed a higher number of studies over the mean value (Figure 3). The Egger regression test suggested a significant association between study size and study effect (*P* value of 0.002). The Duval and Tweedie trim and fill method suggested adding 10 studies to the left side of the funnel plot, which under the random effects model would shift the point estimate of the prevalence of cyst shedding of all the studies included in this meta-analysis from 0.77 (CI: 0.68, 0.85) to 0.61 (CI: 0.49, 0.72), improving the estimate. The results of Cochran's *Q* test indicated significant heterogeneous results (*P* value < 0.001) among different studies and the *I* ^2^ statistic determined that 88% of variation across studies was due to significant heterogeneity rather than random chance.

### 3.3. Analysis for the Diarrhoea Outcome

As the data for the diarrhoea outcome were sparse, the analysis done for the shedding outcome could not be performed for the diarrhoea outcome. Therefore, the bivariate analysis was done considering the data as a pool across studies to estimate associations rather than accounting for the correlation between studies as done for the shedding outcome. The bivariate analysis showed that, for the outcome of diarrhoea in exposed animals, the covariates “Isolate Source” and “Experimental Species” were significant (*P* value ≤ 0.1) (Table 7). Due to the smaller number of studies reporting on the outcome of diarrhoea and variability in the reporting of these potential covariates across studies, it was not possible to create a multivariable model for this outcome. The publication bias for diarrhoea outcome was not assessed due to the low number of studies (4 studies) included in this meta-analysis.

## 4. Discussion

This meta-analysis evaluated the effects of multiple experimental covariates on cyst shedding or diarrhoea as indicators of* G. duodenalis* infection. The results obtained in this meta-analysis identified covariates that potentially cause heterogeneity between the outcomes of the dose results experiments and suggest that administering cysts or trophozoites to experimental hosts and the dose administered can all significantly impact the incidence of cyst shedding.

Based on the results of this meta-analysis, it would be appropriate to make the following recommendations for future dose-response experiments on* G. duodenalis* when assessing infection by means of the presence of cyst shedding in experimentally infected animals to make the studies more comparable and increase likelihood of cyst shedding: (i) use cysts to infect the experimental animals rather than trophozoites, (ii) consider the infective stage used (cysts versus trophozoites) and the* Giardia* dose administered together, (iii) consider the animal species used as an experimental host and dose together, and (iv) taking this into account, consider using mice as experimental hosts rather than gerbils, rats, hamsters, dogs, cats, and cattle. These parameters should be considered when designing experimental dose-response studies, as once the designs of the dose-response studies are more standardized, they will provide better information and more comparable results for more accurate risk assessments that consider infection as the outcome.

In the case of assessing infection by means of the presence of diarrhoea in experimentally infected animals, more experimental studies in animal models should be conducted, as not enough studies have been reported to obtain estimates of the effect of different experimental parameters on diarrhoea in individual animals. It should be noted that while the diarrhoea outcome is of clinical relevance, the presence of asymptomatic infected individuals is a limitation of using diarrhoea to represent infectious status. Based on the bivariate analyses, it would be appropriate to consider and report the following in future dose-response experiments: (i) the assemblage being inoculated into the experimental host, (ii) original source of the cysts being inoculated, (iii) whether cysts or trophozoites were subject to any passage before inoculation into the experimental host or not, (iv) animal species used as experimental hosts, (v) age of the experimental host, and (vi) the administration route used to inoculate the cysts or trophozoites into the experimental host. These studies will provide better information and more comparable results for risk assessments that consider illness as an outcome.

Interestingly, the significant “*Giardia* Dose by Experimental Species” interaction suggests that an increase of one in log of dose administered has a larger impact on cyst shedding in “Mice” than “Gerbils” as experimental hosts, indicating a larger difference between the experimental host-groups as the administered dose increases. But, an increase of one in log of dose administered had a protective impact on cyst shedding in “Other Rodents” compared to “Gerbils” as experimental hosts. Nevertheless, differences in cyst shedding among experimental animal hosts may depend on the dose of* Giardia *administered, and it is advisable to consider these two variables together when designing experimental studies. In addition, it is important to take into consideration that some species might vary in their susceptibilities to different* G. duodenalis *assemblages. For instance, dogs are the only species that have been reported to be susceptible to* G. duodenalis *assemblage C and D, cats to assemblage F, hoofed livestock to assemblage E, and rats to assemblage G [1, 3, 12], while a wide variety of animals, such as cattle, dogs, cats, rodents, and other wild animals and humans are susceptible to* G. duodenalis* assemblage A [1, 3, 12]. Thus, choosing to use mice, other rodents, or other animals as experimental hosts would be appropriate only for some study objectives, such as evaluating the infectivity of the different* G. duodenalis* assemblagesby means of cyst shedding.

Pathogen shedding patterns for newborns, weanlings, and adults can be quite different across host species. For instance, it has been reported that weanling calves generally lack a strong specific humoral immune response to* G. duodenalis* infection, while newborns may be protected by the anti-*Giardia* activity of colostrums [13], indicating that weanlings may be more susceptible to* Giardia *infection than newborns or adults. Unfortunately, in this meta-analysis there were not enough studies to analyse newborn and weanling animals separately, thus both categories had to be merged and analyzed as “Young.” Based on the meta-analysis results, young animals were not statistically different from adult animals with regard to cyst shedding. This finding is in contrast to numerous studies that reported the prevalence and cyst excretion peaks in young animals [14–16]. However, our results from this meta-analysis are in concordance with the findings reported by Hewlett et al. [17], where young mongrel dogs were not found to be more susceptible than adults, and Woo and Paterson [18] where adult and young dogs and cats did not present with infection after being challenged with 300000 cysts. The data available for this meta-analysis were unfortunately inadequate to provide estimates to determine how the dose and the choice of the age of the experimental species impact the odds of cyst shedding.

The multivariable model suggested that inoculating the experimental host with cysts would increase the likelihood of cyst shedding compared to inoculation with trophozoites. However, the model also indicated that an increase of one log in dose had less of an impact on cysts than it did on trophozoites. Therefore, differences by using cysts versus trophozoites for host infection depend on the Giardia dose used, and it is advisable to consider these two variables together when designing experimental studies. Results of the multivariable model also suggest that when a higher dose of trophozoites is administered to gerbils, the odds of cyst shedding increase. Numerous studies in which different doses of Giardia cysts or trophozoites have been administered to animal models have been published. However, whether the infectious dose may contribute to symptom variability is still unclear [1]. Studies have shown that the infectivity ratio is directly related with the number of cysts inoculated to the experimental host [19, 20]. Nevertheless, all of these reports were individual studies; therefore, there is the possibility that those results were influenced by the experimental design or other factors. Among the strengths of meta-analyses is that it provides an overall estimate of an association or effect based on a number of independent scientific studies and explores the variation in the observed effect across studies, thus obtaining a gain in statistical power to detect effects [21]. The result obtained in the multivariable model indicated that when a higher dose is administered, the odds of having cyst shedding increases.

Information for the variables “Assemblage,” “Isolate Source,” and “Storage Time” was scarce and thus the association between these variables and the shedding outcome could not be assessed. Passage of cysts or trophozoites used to infect experimental hosts was identified as a risk factor for cyst shedding in the bivariate analysis. However, information on this variable was not reported for all studies, which made it challenging to consider it jointly in a multivariable model. Once more experimental studies providing information regarding these variables are published, it would be beneficial to evaluate the association with the shedding outcome and incorporate them in the multivariable model “Experimental Species,” “Age,” “Infective Stage,” and “*Giardia* Dose” to ascertain the independent effect of these possible covariates on the presence of cyst shedding.

This meta-analysis showed evidence of possible publication bias for the cyst shedding outcome. This finding can be explained by the fact that smaller studies are more likely to be published if they have larger than average effects, which makes them more likely to meet the criterion for statistical significance [22]. The addition of studies by the trim and fill procedure improved the point estimate of the prevalence of cyst shedding of all the studies included in this meta-analysis. However, the adjusted estimate is similar to the original effect and thus indicates that the reported trends may be valid. Caution is advised when interpreting these results because there is evidence of high heterogeneity among the studies, thus precluding a full evaluation of publication. These findings suggest that it is important to publish those studies that have negative or nonsignificant results as well those that have significant or positive results in order to reduce or avoid the bias.

Based on the results of this meta-analysis, it is crucial that more experimental studies in animal models are conducted to assess infectivity of* Giardia* by means of cysts shedding and diarrhoea. These studies would provide useful data for risk assessments that consider either infection or illness as an outcome.

## 5. Conclusions

When assessing* G. duodenalis* infection using a cyst shedding outcome measure, this study suggests that differences among the animal species used as experimental hosts depend on the dose of* Giardia* administered. It is therefore advisable to consider these two variables (host and dose) together when designing experimental studies. Taking this into account, mice appear to be the most appropriate animal model in which to assess infection when using a cyst shedding outcome, as they were more likely to shed cysts than other animal species.

Young and adult animals were similarly likely to shed cysts. Nevertheless, additional studies are needed for increased statistical power to ascertain effects of the log dose increment for different age groups on the presence of cyst shedding.

For considering whether cysts or trophozoites are used to challenge the experimental hosts to assess infection by means of cyst shedding, the multivariable analysis results suggest that it would be more appropriate to use cysts. However, it also indicated that an increase of one log dose has less of an impact for cysts than for trophozoites; thus, differences between inoculating cysts or trophozoites in the cyst shedding depend on the dose administrated. As expected, administering higher doses of cysts or trophozoites increases the odds of cyst shedding.

When using a diarrhoea outcome measure in experimental studies, the source of the isolate and species of experimental animal host should be considered when designing experimental studies. As additional studies are published, greater power will be possible to distinguish individual and joint effects of the identified covariates.

## Figures and Tables

**Table 1:** Exclusion criteria for studies and number of articles excluded from the *Giardia duodenalis *meta-analysis.

Exclusion criteria	Articles excluded
(1) *Giardia *different than *G. duodenalis*, * G. intestinalis,*or *G. lamblia *were used	19
(2) Infection was assessed only by histology on tissue sections	6
(3) Number of animals with diarrhoea or shedding was not reported	4
(4) Animal study groups were subject to treatments other than the single inoculation/single outcome design used in this meta-analysis	3
(5) Infection was assessed by cyst shedding and histology together and it was not possible to distinguish how many animals were shedding	4
(6) Experimental dose response was measured in humans only	2
(7) Study was not an experimental dose response experiment	1
(8) Cyst dose administered to the experimental host was not provided or a range was provided	1
(9) Number of animals used was not provided	1

**Table 2:** Studies included for *Giardia duodenalis *meta-analysis of diarrhoea (D) and shedding (S) outcomes (chronological order).

Reference	Year	Outcome	Sample size (number of animals)								
			IS^1^	P^2^	ST^3^	ES^4^	A^5^	IS^6^	GD^7^	AR^8^	DM^9^
[23]	1978	S^10^	20	20	20	20	20	20	20	20	20
[24]	1979	S^10^	47	47	NR^11^	47	47	47	47	47	NR^11^
[17]	1982	S^10^	11	11	11	11	11	11	11	11	NR^11^
[19]	1982	S^10^	150	150	150	150	150	150	150	150	150
[25]	1984	S^10^	49	49	NR^11^	49	49	49	49	49	NR^11^
[26]	1985	S^10^	25	25	14	25	NR^11^	25	25	25	25
[18]	1986	S^10^	60	60	NR^11^	60	49	60	60	60	60
[27]	1988	S^10^	204	204	107	204	204	204	204	204	204
[28]	1989	S^10^	10	10	NR^11^	10	NR^11^	10	10	10	10
[29]	1990	S^10^	75	75	NR^11^	75	75	75	75	75	75
[30]	1991	S^10^	NR^11^	NR^11^	NR^11^	10	10	10	10	10	10
[31]	1991	S^10^	109	109	109	109	109	109	109	109	109
[32]	1992	S^10^	NR^11^	14	NR^11^	14	14	14	14	14	14
[33]	1993	S^10^	62	62	NR^11^	62	62	62	62	62	62
[34]	1994	S^10^	4	16	NR^11^	16	16	16	16	NR^11^	16
[35]	1996	S^10^	10	10	10	10	10	10	10	10	10
[36]	1997	S^10^	NR^11^	10	NR^11^	10	10	10	10	10	10
[37]	2002	S^10^	NR^11^	94	NR^11^	94	94	94	94	94	94
[38]	2005	S^10^	56	56	NR^11^	56	56	56	56	56	56
[39]	2006	S^10^	NR^11^	6	NR^11^	6	6	6	6	6	6
[40]	2007	S^10^	97	97	97	97	97	97	97	97	97
[41]	2007	S^10^	6	6	NR^11^	6	6	6	6	6	6
[42]	2008	S^10^	NR^11^	12	NR^11^	12	12	12	12	12	12
[43]	2008	S^10^	NR^11^	60	NR^11^	60	60	60	60	60	60
[44]	2010	S^10^	40	40	NR^11^	40	40	40	40	40	40
[45]	2010	S^10^	33	33	33	33	33	33	33	33	33
[46]	1995	S and D^12^	70^13^, 28^14^	70^13^, 28^14^	30^13^, 8^14^	70^13^, 28^14^	70^13^, 28^14^	70^13^, 28^14^	70^13^, 28^14^	40^13^, 20^14^	70^13^, NA^15^
[47]	1995	S and D^12^	52^13^, 30^14^	52^13^, 30^14^	22^13^, NR^11^	52^13^, 30^14^	52^13^, 30^14^	52^13^, 30^14^	52^13^, 30^14^	52^13^, 30^14^	52^13^, NA^15^
[48]	1997	S and D^12^	10^16^	10^16^	NR^11^	10^16^	10^16^	10^16^	10^16^	10^16^	NA^15^
[49]	2010	S and D^12^	14^16^	14^16^	NR^11^	14^16^	14^16^	14^16^	14^16^	14^16^	NA^15^

^1^Isolate Source, ^2^Passage, ^3^Storage Time (weeks), ^4^Experimental Species, ^5^Age, ^6^Infective Stage, ^7^ *Giardia* Dose, ^8^Administration Route, ^9^Detection Method, ^10^Shedding outcome, ^11^Information was not reported in study; thus no individual data were included, ^12^Shedding and Diarrhoea outcome, ^13^Number of individuals for shedding outcome, ^14^Number of individuals for diarrhoea outcome, ^15^Does not apply, ^16^Number of individuals for both outcomes.

**Table 3:** Classification of categorical variables for both *Giardia duodenalis *cyst shedding and diarrhoea outcomes.

Variable	Shedding outcome			Diarrhoea outcome		
	Classification of variables^1^	Number of studies	Number of individuals	Classification of variables^1^	Number of Studies	Number of individuals
Assemblage	A	3	148	A and E	1	14
	B	1	49			
	E	1	6			
	A and E	1	14			

Isolate Source	Humans Other Than Humans	19 4	1128 86	Humans Other Than Humans	1 3	28 54

Passage	No Yes	17 16	755 667	No Yes	2 2	24 58

Storage Time	<1 week ≥1 week	10 3	420 183	<1 week	1	8

Experimental Species	Mice Other Animals Other Rodents Gerbils	7 8 4 15	180 87 202 963	Other Animals Gerbils	2 2	24 58

Age	Adult Young	12 18	458 828	Newborn Weanling	2 2	24 58

Infective Stage	Cysts Trophozoites	20 18	1021 411	Cysts Trophozoites	3 2	64 18

Administration Route	Gastric Intubation Other Than Gastric Intubation	6 23	233 1153	Gastric Intubation Other Than Gastric Intubation	2 2	50 24

Detection Method	Hemocytometer Microscopy Flotation Technique	15 3 9	749 53 523	Not analyzed		

^1^Categories not listed indicate that there were no animals from those categories in the studies.

**Table 4:** Criteria used to determine age categories for experimental species (alphabetical order).

Animal species	Criteria used		
	Newborn	Weanling^1^	Adult
Cattle	<21 weeks	21–64 weeks [50]	≥65 weeks [51]
Cats	<8 weeks	8–25 weeks [52]	≥26 weeks [51]
Dogs	<8 weeks	8–29 weeks [53]	≥30 weeks [51]
Gerbils	<4 weeks	4–7 weeks [54]	≥8 weeks [55, 56]
Hamsters	<3 weeks	3 weeks [57]	>3 weeks
Mice	<3 weeks	3 weeks [58]	>3 weeks [51]
Rabbits	<4 weeks	4 weeks–17 weeks [59, 60]	≥18 weeks [51]
Rat	3 weeks	3–7 weeks [61]	≥8 weeks[51]

^1^Weanling was merged with the newborn category to create the classification “Young” in the “Age” variable for the cyst shedding outcome.

**Table 5:** Bivariate analysis results for risk factors associated with *Giardia* cyst shedding outcomes.

Variable^1 ^	*P*-value	Statistical association between variable and cyst shedding (Yes/No)^2^
Passage	<0.0001^3^	Yes
Storage Time	0.9269^3^	No
Experimental Species	<0.0001^3^	Yes
Age	0.0023^3^	Yes
Infective Stage	<0.0001^3^	Yes
*Giardia* Dose	0.0061^4^	Yes

^1^Data sparseness prevented analytic calculations for Isolate Source, Administration Route and Detection Method.

^2^Significant level ≤0.2.

^3^Mantel-Haenzel bivariate analyses analysis taking into account the correlation between studies.

^4^GLIMMIX bivariate analyses.

**Table 6:** Multivariable generalized linear mixed model showing risk factor associations for *Giardia duodenalis* cyst shedding outcomes^1^.

Variable	Categories	Odds ratio estimate	95% Wald confidence limits	*P* value
Experimental Species	Gerbils^2^			
	Mice	0.16^3^	(0.003, 8.87)	0.37
	Other Animals	0.03^4^	(<0.001, 5.05)	0.18
	Other Rodents	4.30^5^	(0.10, 183.05)	0.45

Age	Young^2^			
	Adult	0.36	(0.05, 2.43)	0.30

Infective Stage	Trophozoites^2^			
	Cysts	5.02^6^	(2.63, 9.56)	<0.001^12^

*Giardia* Dose	Not applied (continuous variable)	2.67^7^	(1.81, 3.94)	<0.001^12^

Administration Route	Gastric Intubation^2^			
	Other Than Gastric Intubation	10.39	(0.19, 573.61)	0.25

*Giardia* Dose by Experimental Species	Mice	4.36^8^	(1.59, 11.93)	0.004^12^
	Other Animals	2.12^9^	(0.12, 37.45)	0.61
	Other Rodents	0.09^10^	(0.01, 0.72)	0.02^12^

*Giardia* Dose by Infective Stage	Cysts	0.57^11^	(0.41, 0.81)	0.002^12^

^1^Pseudo-AIC = 7013.49.

^1^Generalized chi-square statistic and its degrees of freedom (gener. Chi-sq/df) = 0.66.

^2^Corresponds to the reference category.

^3^The odds ratio estimate corresponds to Mice versus Gerbils at the mean log dose.

^4^The odds ratio estimate corresponds to Other Animals versus Gerbils at the mean log dose.

^5^The odds ratio estimate corresponds to Other Rodents versus Gerbils at the mean log dose.

^6^The odds ratio estimate corresponds to Cysts versus Trophozoites at the mean log dose.

^7^The odds ratio estimate corresponds to an increase of one in log dose in Gerbils with Trophozoites.

^8^The odds ratio corresponding to an increase of one in log dose in Mice is 4.36 times that in Gerbils.

^9^The odds ratio corresponding to an increase of one in log dose in Other Animals is 2.12 times that in Gerbils.

^10^The odds ratio corresponding to an increase of one in log dose in Other Rodents is 0.09 times that in Gerbils.

^11^The odds ratio corresponding to an increase of one in log dose in Cysts is 0.57 times that in Trophozoites.

^12^Risk factors with statistically significant results.

**Table 7:** Bivariate analysis results for risk factors associated with diarrhoea outcome after *Giardia* infection.

Variable^1^	*P* value	Statistical association between variable and presence of diarrhoea (Yes/No)^2^
Isolate Source	<0.0001^3^	Yes
Passage	0.35^3^	No
Experimental Species	0.07^3^	Yes
Age	0.35^3^	No
Infective Stage	0.25^3^	No
*Giardia* Dose	0.25^4^	No
Administration Route	0.32^3^	No

^1^Data sparseness prevented analytic calculations for Storage Time.

^2^Significant level ≤0.2.

^3^Mantel-Haenzel bivariate analyses not taking into account the correlation between studies.

^4^GLIMMIX bivariate analyses.
